# A novel small molecule STAT3 inhibitor, LY5, inhibits cell viability, colony formation, and migration of colon and liver cancer cells

**DOI:** 10.18632/oncotarget.7338

**Published:** 2016-02-11

**Authors:** Chongqiang Zhao, Wenlong Wang, Wenying Yu, David Jou, Yina Wang, Haiyan Ma, Hui Xiao, Hua Qin, Cuntai Zhang, Jiagao Lü, Sheng Li, Chenglong Li, Jiayuh Lin, Li Lin

**Affiliations:** ^1^ Division of Cardiology, Department of Internal Medicine, Tongji Hospital, Tongji Medical College, Huazhong University of Science and Technology, Wuhan, P.R. China; ^2^ Division of Cardiology, Tianjin First Center Hospital, Tianjin, P.R. China; ^3^ Division of Pediatric Intensive Care Unit, Pediatric Cardiac Center, Fuwai Hospital, Chinese Academy of Medical Sciences and Peking Union Medical College, Beijing, P.R. China; ^4^ Division of State Key Laboratory of Natural Medicines, China Pharmaceutical University, Nanjing, P.R. China; ^5^ Center for Childhood Cancer and Blood Diseases, The Research Institute at Nationwide Children's Hospital, Department of Pediatrics, College of Medicine, The Ohio State University, Columbus, OH, USA; ^6^ Division of Gastroenterology, Departments of Internal Medicine, Tongji Medical College, Huazhong University of Science and Technology, Wuhan, P.R. China; ^7^ Departments of Geriatrics, Tongji Hospital, Tongji Medical College, Huazhong University of Science and Technology, Wuhan, P.R. China; ^8^ Division of Medicinal Chemistry and Pharmacognosy, College of Pharmacy, The Ohio State University, Columbus, OH, USA

**Keywords:** LY5, STAT3, colon cancer, liver cancer

## Abstract

Signal Transducer and Activator of Transcription 3 (STAT3) is persistently activated in human liver and colon cancer cells and is required for cancer cell viability, survival and migration. Therefore, inhibition of STAT3 signaling may be a viable therapeutic approach for these two cancers. We recently designed a non-peptide small molecule STAT3 inhibitor, LY5, using in silico site-directed Fragment-based drug design (FBDD). The inhibitory effect on STAT3 phosphorylation, cell viability, migration and colony forming ability by LY5 were examined in human liver and colon cancer cells. We demonstrated that LY5 inhibited constitutive Interleukin-6 (IL-6)-induced STAT3 phosphorylation, STAT3 nuclear translocation, decreased STAT3 downstream targeted gene expression and induced apoptosis in liver and colon cancer cells. LY5 had little effect on STAT1 phosphorylation mediated by IFN-γ. Inhibition of persistent STAT3 phosphorylation by LY5 also inhibited colony formation, cell migration, and decreased the viability of liver cancer and colon cancer cells. Furthermore, LY5 inhibited STAT3 phosphorylation and suppressed colon tumor growth in a mouse model *in vivo*. Our results suggest that LY5 is a potent STAT3 inhibitor and may be a potential drug candidate for liver and colon cancer therapy.

## INTRODUCTION

Primary liver cancer is the fifth most common human cancer, has a high incidence rate and poor prognosis. Hepatocellular carcinoma (HCC) accounts for more than 85% of all primary liver cancers, with a 5-year survival rate of 9% and a median survival time of less than 1 year. Recent data indicate that the mortality of HCC in China has been increasing [1]. Colorectal carcinoma is the third leading cause of cancer-related deaths in the United States. According to the American Cancer Society, there will be an estimated 134,490 new cases and 49,190 deaths due to colorectal cancer in the United States in 2016 [2]. Therefore, there is a critical need for better approaches to treatment for these types of cancers.

The cellular mechanisms contributing to colorectal and liver cancer are not well understood but involve signaling protein dysregulation, including the persistent activation of STAT3. STAT3 is a transcription factor and oncogenic driver that promotes malignancy [3]. In contrast to normal cells where activation of STAT3 is only transient due to a host of endogenous protein regulators (e.g., PIAS, SOCS), aberrant and constitutive activation of STAT3 has been detected in a wide variety of human cancers, including solid tumors as well as blood malignancies [4]. Phosphorylation of Tyr705 in STAT3 leads to dimerization, nuclear translocation, recognition of STAT3-specific DNA binding elements and up-regulation of various STAT3 downstream target genes, such as Bcl-xl, Bcl-2, Survivin, c-Myc, cyclin D1, and others [5]. Interestingly, no obvious deleterious effects were observed when STAT3 antisense therapy was used to deplete it from normal cells in mice [6]. In contrast to STAT3 activation in normal cells, constitutive activation of STAT3 stimulates tumor cell proliferation and migration, mediates immune evasion, promotes angiogenesis, and confers resistance to apoptosis induced by conventional therapies [7, 8]. The finding that persistent activation of STAT3 is dispensable for normal cells but is essential for the development and progression of tumors strongly suggests that STAT3 could be a potential therapeutic target for cancers. Growing evidence has shown that blockade of constitutively activated STAT3 can cause apoptosis and decrease proliferation and cell migration *in vitro* [9–12], inhibit tumor growth *in vivo* [13–16], as well as enhance the sensitivity to chemotherapy and radiotherapy [17–20].

Persistent activation of STAT3 signaling is frequently detected in colon [21] and liver cancers [22, 23]. Constitutive STAT3 activation in colorectal cancer cells is correlated with invasion, survival, and growth of colorectal cancer cells in a colorectal tumor model in mice *in vivo* [24, 25]. Persistent STAT3 activation in liver cancer cells is also associated with invasion, survival, proliferation, and tumorigenesis of liver cancer cells [9, 10, 14, 26]. These reports indicate that STAT3 is one of the major oncogenic pathways activated in colorectal and liver cancers and can serve as a viable therapeutic target for these two cancer types. To directly target persistent STAT3 signaling in cancer cells, we recent developed a novel small molecular STAT3 inhibitor LY5, which was derived from LLL12 by an in silico site-directed Fragment-based drug design [27]. Fragment-based drug design method was used to identify the fragments from several known STAT3 inhibitors which target the STAT3 Src homolog 2 (SH2) domain. STAT3 fragment libraries were built from several known inhibitors and divided into two specific sub-libraries for the pTyr705 site and the side pocket site based on the docking poses of the inhibitors to the STAT3 SH2 domain. During LY5 drug design, we chose the fragment for the pTyr 705 site of LLL12 which had the lowest IC50 among the known nonpeptidomimetic small inhibitors and the fragment for the side pocket of ISS219. In order to maintain their poses in the binding sites and reduce synthesis difficulty, we chose dimethyl amine as the linker and merged the two chosen fragments. The aforementioned fragments that specifically bound to each of the two STAT3 SH2 binding sites, pTyr705 and the side pocket, were selected and linked to form the novel compound, LY5, whose formal chemical name is 5, 8-dioxo-6-(pyridin-3-ylamino)-5, 8-dihydronaphthalene-1-sulfonamide [27].

We evaluated the inhibitory effects of LY5 on constitutive and inducible STAT3 phosphorylation and the expression of its downstream target genes in colon cancer cells and liver cancer cells. Furthermore, we demonstrated that blockade of persistent STAT3 signaling inhibited proliferation, cell migration and colony formation, as well as induced apoptosis in liver and colon cancer cells. Moreover, LY5 suppressed colon tumor growth in a mouse xenograft model.

## RESULTS

### LY5 inhibited persistent STAT3 phosphorylation and induced apoptosis in colon cancer cells

**Figure 1:**
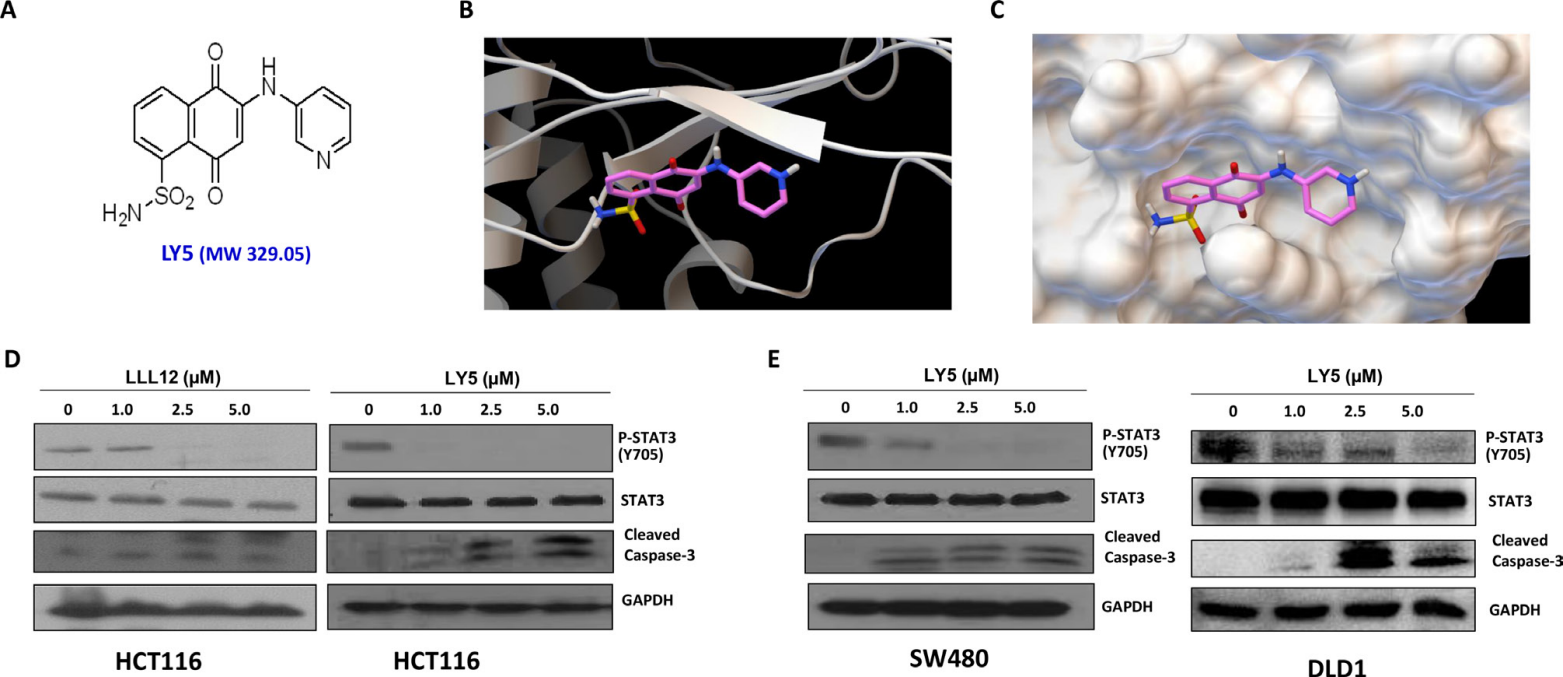
LY5, a novel STAT3 inhibitor decreased constitutive phosphorylation of STAT3 in colon cancer cells (**A**) Chemical structure of LY5. (**B**) and (**C**) The docking mode of LY5 and STAT3 crystal structure (PDB:1BG1). LY5 can bind to the pTyr705 and side pocket sites. (**D**) LY5 and LLL12 (1.0, 2.5, and 5 μM) inhibited STAT3 phosphorylation and induce apoptosis in HCT116 colon cancer cells. (**E**) LY5 (1.0, 2.5, and 5 μM) inhibited STAT3 phosphorylation and induced cell apoptosis in SW480 and DLD1 human colon cancer cells.

LY5 (Figure 1A) was docked into the crystal structure of STAT3 protein by software Autodock4. The structure in ribbon and surface mode demonstrating how LY5 interacts with STAT3 is shown in Figure 1B and 1C. LY5 formed three hydrogen bonds with the STAT3 SH2 domain, with residues Arg609, Ser613 and Ser636. It was predicted that LY5 could fit into the two major binding sites, the pTyr705 and the side pocket site, so that it could inhibit both STAT3 phosphorylation and dimerization. To confirm this, we first examined whether LY5 inhibits constitutive STAT3 phosphorylation in colon and liver cancer cells. HCT116 colon cancer cells were treated with LY5. LLL12, a previously developed STAT3 inhibitor was included as a comparison. LY5 inhibited persistent STAT3 phosphorylation at lower concentrations (1.0 μM) than LLL12 (Figure 1D). LY5 exhibited greater potency than LLL12 when dissolved in the same DMSO concentrations (Supplementary Figure S1, Supplementary Table S1). Therefore, LY5 has better water solubility than LLL12. After treatment with LY5 for 24 hours, LY5 also inhibited persistent STAT3 phosphorylation and induced cleaved capase-3, a hallmark of apoptosis, in SW480 and DLD1 colon cancer cells (Figure 1E).

### LY5 suppressed STAT3 phosphorylation, decreased STAT3 downstream target genes expression and induced apoptosis in liver cancer cells

**Figure 2:**
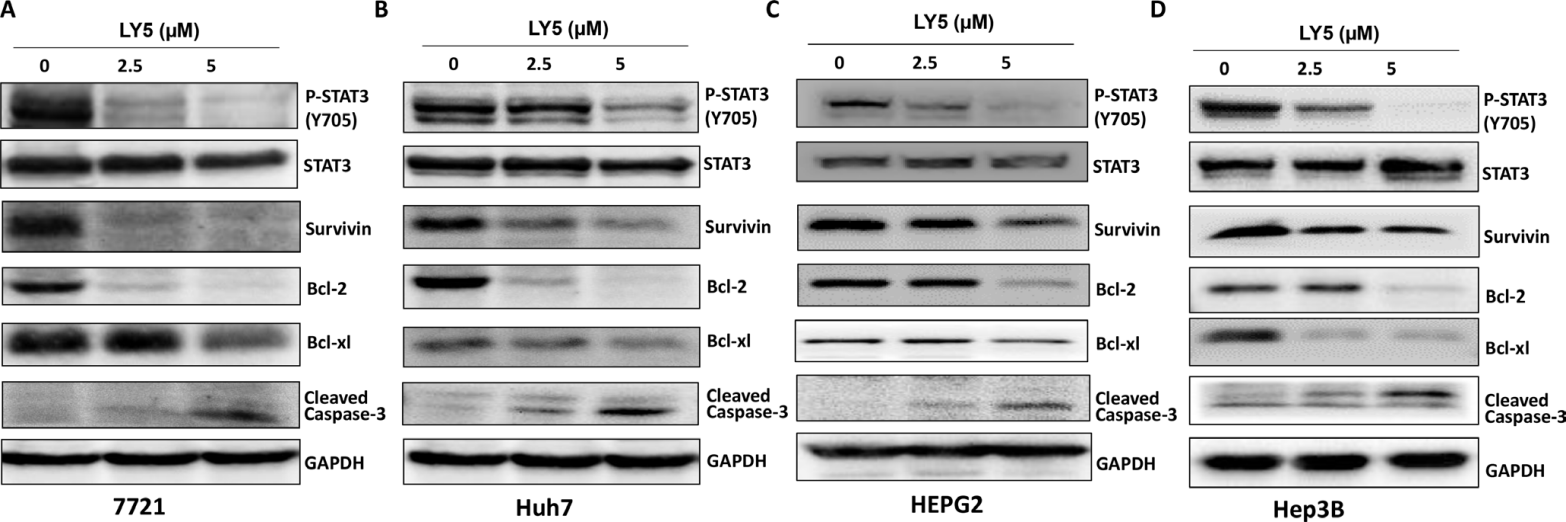
LY5 suppressed STAT3 phosphorylation, decreased STAT3 downstream target genes expression and induce apoptosis in 7721 (A), Huh7 (B), HEPG2 (C) and Hep3B (D) liver cancer cell lines The expression of STAT3 downstream genes, Survivin, Bcl-2, Bcl-xl, was detected by western blot after treated with LY5 for 24 hours.

Persistent STAT3 phosphorylation in SSMC 7721, Huh7, HEPG2, and Hep3B liver cancer cell lines were inhibited after treatment with LY5 for 24 hours (Figure 2A–2D). Furthermore, LY5 had no effect on the overall expression of STAT3 (Figure 2A–2D).

The expression of STAT3 target genes, Survivin, Bcl-2 and Bcl-xl, were decreased in SSMC 7721, Huh7, HEPG2 and Hep3B liver cancer cells after treatment with LY5 as examined by Western Blot (Figure 2A–2D). Our results also showed that LY5 induced the cleavage of caspase-3, indicating apoptosis in these liver cancer cells (Figure 2A–2D).

### LY5 inhibited IL-6-induced STAT3 phosphorylation and did not affect IFN-γ induced STAT1 phosphorylation

**Figure 3:**
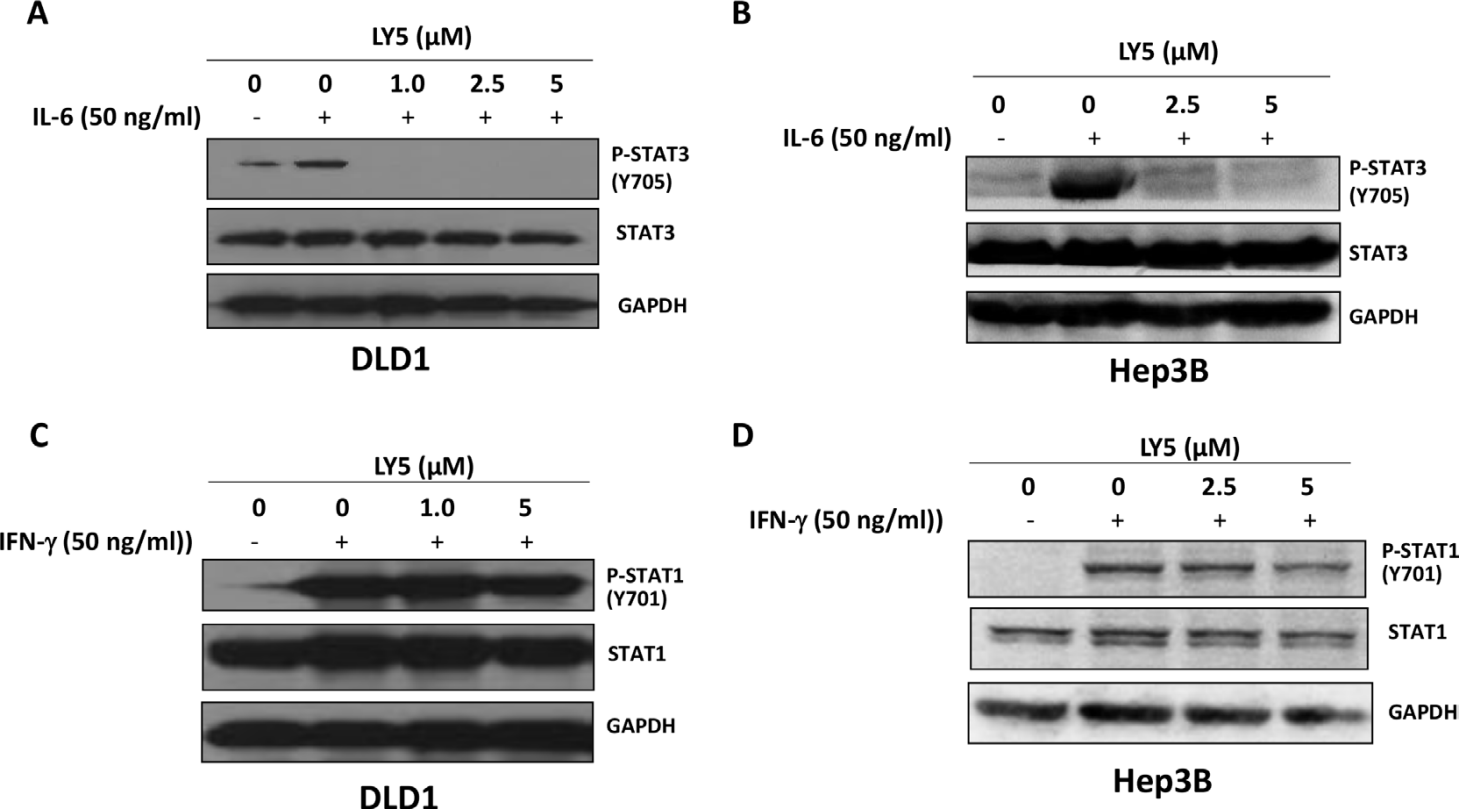
LY5 inhibited STAT3 phosphorylation induced by IL-6 LY5 inhibited STAT3 phosphorylation induced by IL-6 in (**A**) DLD1 colon cancer and (**B**) Hep3B liver cancer cells. LY5 did not inhibit STAT1 phosphorylation induced by IFN-γ in (**C**) DLD1 colon cancer and (**D**) Hep3B liver cancer cells. After 2 hours of LY5 pre-treatment, cells were stimulated by IL-6 (50 ng/mL) or IFN-γ (50 ng/mL) for 30 mins. The cells were harvested and analyzed for STAT3 and STAT1 phosphorylation by western blot as described in Materials and Methods.

Our results showed that IL-6 stimulated STAT3 phosphorylation on tyrosine 705 in DLD1 and Hep3B cancer cells. The induction of STAT3 phosphorylation by IL-6 was inhibited by LY5 in a dose-dependent manner (Figure 3A and 3B). Thus, LY5 is capable of inhibiting IL-6 induced STAT3 phosphorylation. Total STAT3 was not affected by the treatment of LY5 (Figure 3A and 3B). We further investigated whether LY5 inhibits STAT1 phosphorylation mediated by IFN-γ. The results showed that the phosphorylation and expression of STAT1 were not inhibited significantly in these cells with the same concentrations of LY5 that inhibited the phosphorylation of STAT3 (Figure 3C and 3D). These data indicated that LY5 selectively inhibits STAT3 phosphorylation stimulation by IL-6.

### LY5 inhibited cell viability and decreased colony formation

**Figure 4:**
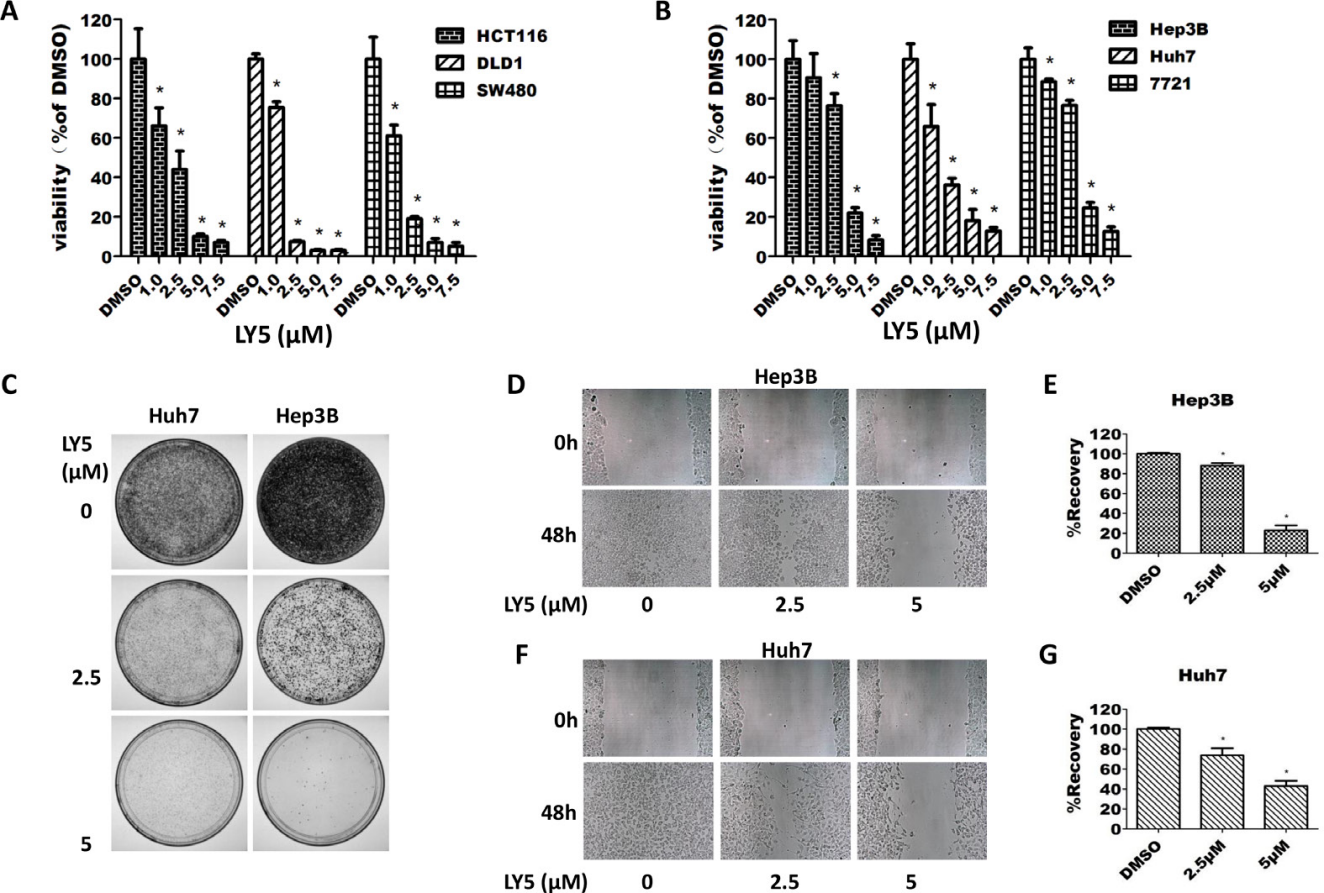
LY5 inhibited the cell viability, migration and colony formation in cancer cells (**A**) Human colon cancer cell lines HCT116, DLD1, and SW480 were cultured with DMSO or LY5 (1, 2.5, 5, and 7.5 μM) for 24 hours and cell viability was measured by MTT viability assays (3 replicates per condition). (**B**) Human liver cancer cell lines Hep3B, Huh7 and 7721 were cultured with DMSO or LY5 (1, 2.5, 5, and 7.5 μM) for 24 hours and cell viability was measured by MTT viability assays (3 replicates per condition). (**C**) LY5 inhibited colony formation in Hep3B and Huh7 human liver cancer cell lines. Wound healing assay for cell migration was carried out with LY5 (2.5 and 5 μM) in Hep3B (**D**) and Huh7 (**F**) liver cancer cell lines as described in Materials and Methods. When the wound in the control was closed, the inhibition of cell migration was assessed by using the ImageJ software (**E** and **G**), the % of wound healing was calculated using the formula: 100 - (final area/initial area × 100%).

In HCT116, SW480 and DLD1 colon cancer cells, treatment with LY5 for 24 hours resulted in a dramatic decrease in cell viability in a dose-dependent manner (Figure 4A). A dose-dependent inhibition of cell viability in Hep3B, Huh7, and SMCC 7721 liver cancer cells was also observed after treatment with LY5 for 24 hours (Figure 4B). The IC50 values for LY5 were 1.637 to 3.347 μmol/L in liver cancer cells and 1.235 to 1.690 μmol/L in colon cancer cells (Supplementary Table S2).

Our results demonstrated that LY5 remarkably reduced the colony formation capacity in Huh7 and Hep3B liver cancer cell lines (Figure 4C). An MTT assay confirmed that the ability of LY5 to inhibit colony formation is not due to induction of cell death (Supplementary Figure S3A, S3B).

### LY5 inhibited cell migration

We next evaluated the effect of LY5 on cell migration by using wound healing assays in Hep3B and Huh7 liver cancer cells. Our results showed that treatment with 2.5 μM and 5 μM of LY5 caused a dose-dependent decrease in cell migration (Figure 4D–4G). A cell viability assay (MTT) determined that the ability of LY5 to inhibit cell migration does not seem to be due to an inhibition of cell proliferation (Supplementary Figure S3C, S3D).

### LY5 inhibited STAT3 nuclear translocation in liver and colon cancer cells

**Figure 5:**
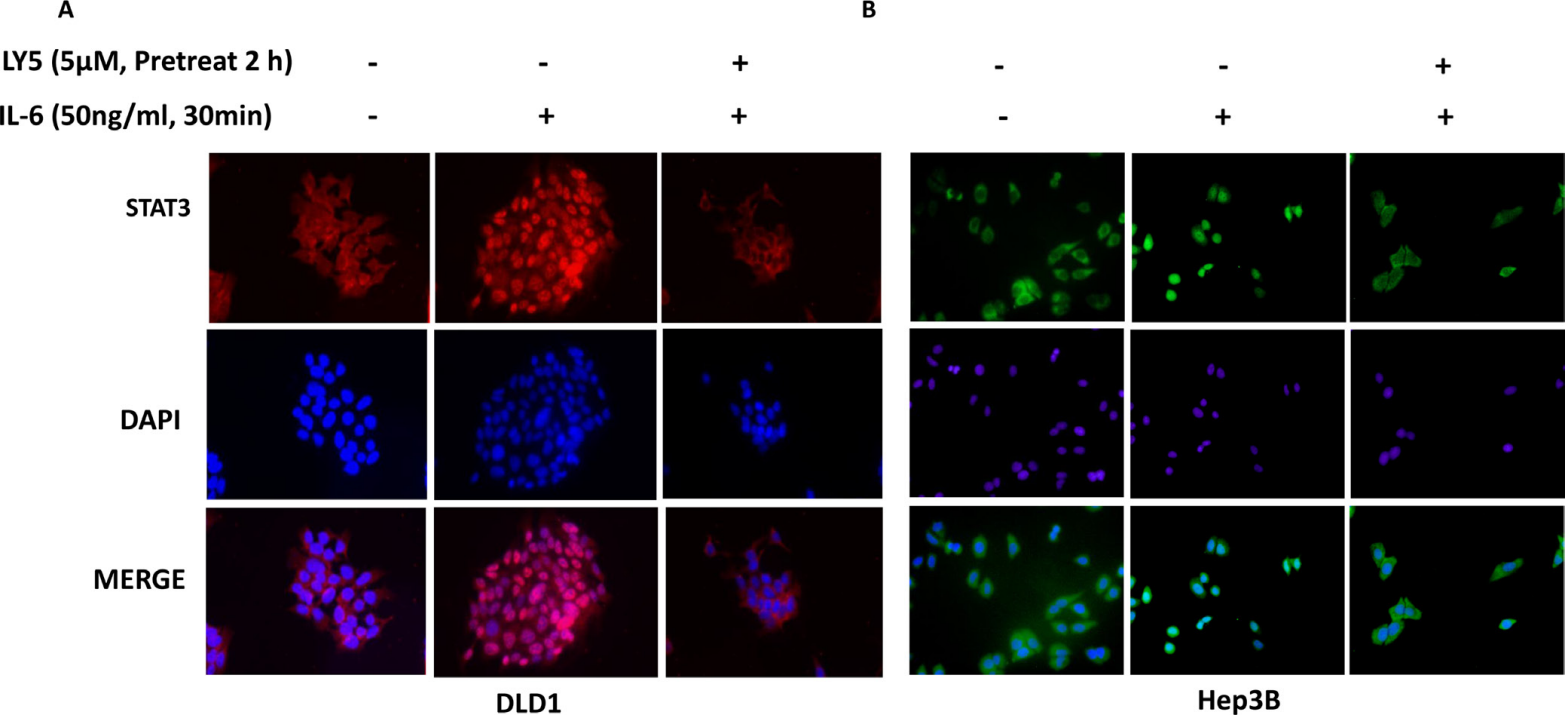
LY5 inhibited STAT3 nuclear translocation induced by cytokine IL-6 After serum-free overnight, DLD1 (**A**) colon and Hep3B (**B**) liver cancer cells were pretreated with LY5 for 2 hours, followed by IL-6 (50 ng/mL) for 30 min, and then processed for STAT3 nuclear translocation detection by immunofluorescence staining.

In Hep3B and DLD1 cells treated with IL-6, STAT3 was phosphorylated and translocated into the nucleus. However, in cells pretreated with LY5 (5 μM), most of the STAT3 was retained in cytoplasm (Figure 5). Additionally, IL-6 induced STAT3 phosphorylation in the nucleus, which was blocked by LY5 pre-treatment in Hep3B liver cancer cells (Supplementary Figure S4). Our data also revealed that p-STAT3 constantly localizes in the nuclei of HCT116, SW480, 7721, Huh7, HEPG2 and Hep3B cells in 10% FBS without IL-6 stimulation. The nuclear localization of p-STAT3 in these cell lines can be inhibited by LY5 (Supplementary Figures S5–S10). Therefore, these results demonstrated that the inhibition of STAT3 phosphorylation by LY5 impaired STAT3 transcriptional function in liver and colon cancer cells by blocking nuclear translocation.

### LY5 suppressed tumor growth of colon cancer cells *in vivo*

**Figure 6:**
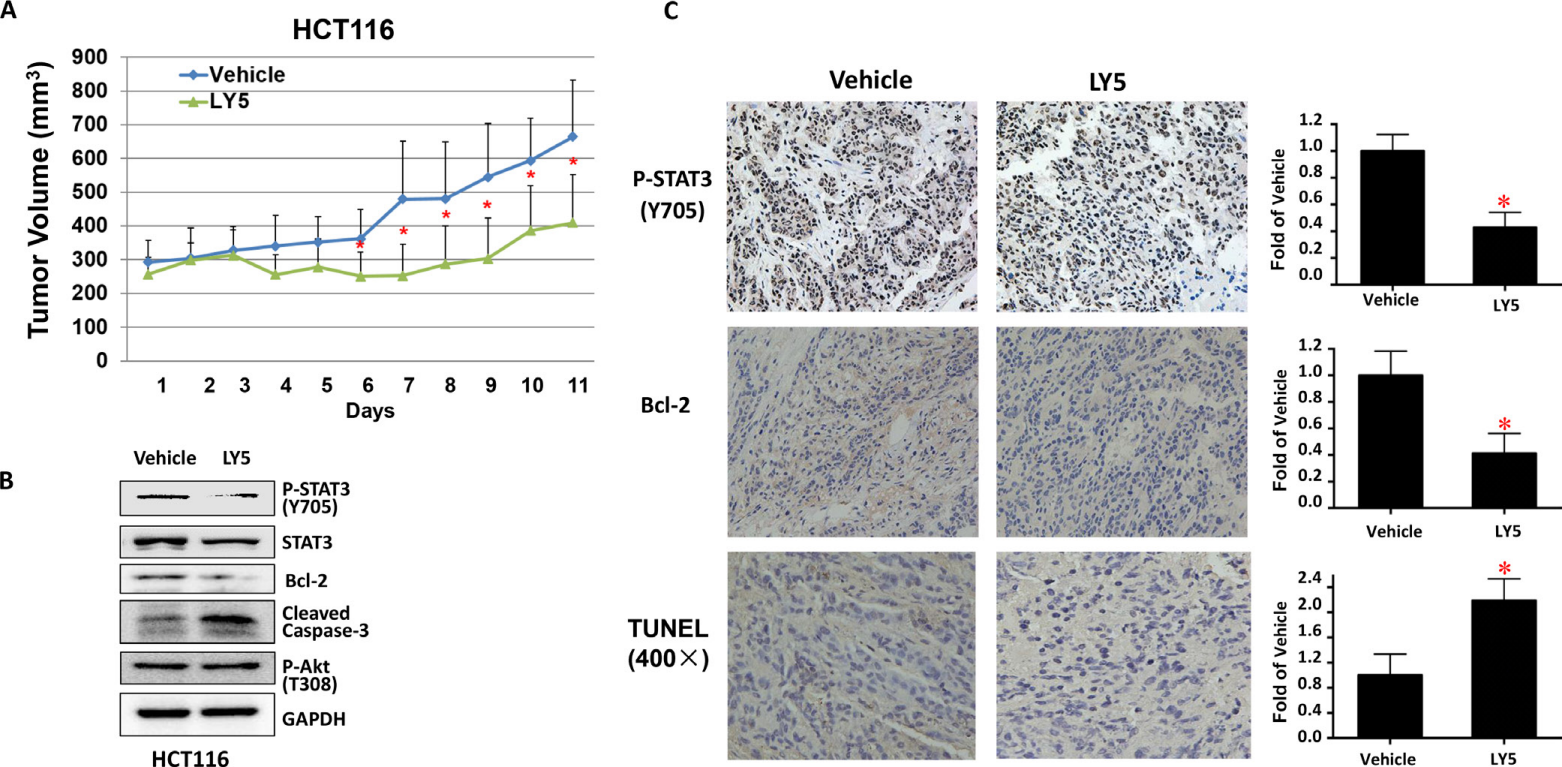
LY5 inhibited tumor growth in mouse xenografts with HCT116 colon cancer cells (**A**) LY5 decreased tumor volumes compared with vehicle group. After the tumor development, the mice were randomized to give daily intraperitoneal dosages of 5 mg/kg of LY5 or vehicle control for 11 days and tumor volumes were determined. (**B**) LY5 inhibited STAT3 but not Akt phosphorylation, decreased Bcl-2 and induced caspase-3 cleavage in mouse xenografts *in vivo*. (**C**) LY5 also inhibited STAT3 phosphorylation, decreased the expression of Bcl-2 as shown by IHC staining and induced apoptosis by using TUNEL assay in xenograft tumors (*p < 0.05).

Finally, we tested the effect of LY5 on suppressing HCT116 colon cancer cells in nude mice xenograft models *in vivo*. As shown in Figure 6A, LY5 suppressed tumor growth compared to the vehicle-treated controls in HCT116 xenografted mice (*P* < 0.05). LY5 inhibited STAT3 but not Akt phosphorylation, decreased BCL-2 and induced caspase-3 cleavage, indicating apoptosis *in vivo* (Figure 6B). LY5 also inhibited STAT3 phosphorylation, decreased the expression of Bcl-2 as shown by IHC staining and induced apoptosis by using TUNEL assay in xenograft tumors (Figure 6C). These results demonstrated that inhibition of STAT3 by LY5 resulted in the suppression of tumor growth in mice, suggesting LY5 might be a potent compound in suppressing tumor growth *in vivo*.

## DISCUSSION

STAT3, a member of the STAT family of transcription factors, is frequently activated in many types of cancers including liver and colon cancers [4, 28, 29]. STAT3 has been classified as an oncogene because activated STAT3 can stimulate oncogenic transformation in cultured cells and tumor formation in nude mice [3, 30]. In contrast, STAT3 deficient fibroblasts were shown to be resistant to transformation by a variety of oncogenes [31, 32]. STAT3 activation results in the expression of downstream target genes, such as cyclin D1, Survivin, Bcl-xl, and Bcl-2, which promote cell proliferation and resistance to apoptosis [28, 30, 33, 34]. In addition, persistent STAT3 signaling also stimulates tumor cell migration, mediates immune evasion, and promotes angiogenesis, which contribute to oncogenesis. The crucial role of STAT3 in cancer progression and tumorigenesis makes STAT3 a viable molecular target for cancer therapy [4, 29, 33].

Previous methods aimed at blocking persistent STAT3 signaling have been reported including phosphopeptide mimics, DNA decoys, dominant-negative STAT3 mutants, STAT3 small interfering RNAs (siRNAs), and anti-sense STAT3 oligonucleotides [27]. In addition, non-peptide small molecule STAT3 inhibitors have been developed, including WP1066 [35–37], S3I-201 [38, 39], STA-21 [40], Stattic [41, 42], and SD-1029 [43]. However, since there are no STAT3 targeting drugs in clinical trials beyond Phase II, the development of more potent and selective compounds that target STAT3 in cancer cells is still needed. We recently developed a novel small molecule STAT3 inhibitor LY5 using in silico site-directed Fragment-based drug design. In this study, we evaluated the inhibitory efficacy of LY5 in liver and colon cancer cells with persistent STAT3 signaling. The results demonstrate that LY5 is capable of inhibiting STAT3 phosphorylation and nuclear translocation. LY5 inhibited STAT3 downstream target genes Bcl-2, Bcl-xl and Survivin, resulting in the induction of apoptosis. In addition to its anti-apoptotic function, STAT3 also stimulates proliferation and cell migration. The results indicated that LY5 inhibited cell viability, colony-forming ability, and cell migration. Water solubility is an important property of synthetic small molecules and for drug development. We found LY5 has better water solubility and a more potent inhibitory effect on cell viability than LLL12, a previously developed STAT3 inhibitor, which LY5 was derived from. In this and other studies, we found LY5 has better water solubility and better *in vivo* PK profile than LLL12. Our previous data have indicated that the potency of LLL12 was higher than that of previously reported STAT3 inhibitors (such as LLL3, WP1066, Stattic, and S3I-201) in human cancer cells [5]. So LY5 may be a good candidate agent having better chance than several other non-peptide small molecule inhibitors mentioned above to suppressing cancer cells by targeting STAT3.

LY5 inhibits persistent STAT3 phosphorylation as well as IL-6-induced STAT3 phosphorylation. Several pro-inflammatory cytokines released by immune cells have been shown to promote cancer cell proliferation and tumor growth and progression. Increased expression of IL-6 is thought to act as a link between chronic inflammation and tumor development [44]. IL-6 stimulates the progression of colon and liver cancers and elevated IL-6 levels are associated with poor prognosis in liver and colon cancers [45–47]. IL-6 stimulates STAT3 phosphorylation and nuclear translocation of STAT3 to promote oncogenesis by inhibiting apoptosis. STAT3 phosphorylation could be blocked by LY5 in a dose-dependent manner. We also examined whether LY5 inhibits STAT1 phosphorylation induced by IFN-γ. Our results showed LY5 did not affect the phosphorylation and expression of STAT1 in these cells suggesting it selectively inhibits STAT3 phosphorylation.

In addition, LY5 suppresses tumor growth in HCT116 colon cancer xenografts mice. These results suggest that LY5 alone or in combination with another chemotherapeutic agent may be effective in suppressing tumor cell growth in cancer patients with constitutive STAT3 signaling. Based on our findings, treatment with LY5 produces both anti-proliferative and pro-apoptotic effects. LY5 has the potential to become a drug candidate for targeting cancer cells with constitutively activated STAT3 due to its ability to inhibit STAT3 phosphorylation and suppress growth. Thus, LY5 deserves to be further developed as a potential drug candidate for liver and colon cancer therapy.

## MATERIALS AND METHODS

### Human liver and colon cancer cell lines

Human liver cancer cell lines (SSMC 7721, Huh7, HEPG2, Hep3B) and colon cancer cell lines (HCT116, SW480, DLD1) were purchased from the American Type Culture Collection and maintained in Dulbecco's modified Eagle's medium/high glucose supplemented with 10% FBS (HyClone) and 1% penicillin/streptomycin. All cancer cell lines were cultured in a humidified 37°C incubator with 5% CO_2_.

### STAT3 inhibitors

Small molecular STAT3 inhibitors LY5 and LLL12 were synthesized in Dr. Chenglong Li's laboratory at the College of Pharmacy, The Ohio State University.

### Cell viability assay

MTT cell viability assay Kits were purchased from Promoter Biotechnology Ltd. Liver and colon cancer cells (5000/well in 96-well plates) were maintained in DMEM supplemented with 10%FBS and 1% penicillin/streptomycin for 24 hours. Then cells were treated with different concentrations of LY5 in triplicate well at 37°C for 24 hours. MTT viability assay was performed according to manufacturer's protocol (Promoter Biotechnology Ltd). The absorbance (A) was read at 570 nm. Cell viability was calculated as follows: (A of LY5 group-A of blank group/A of DMSO group-A of blank group) × 100%.

### Colony formation

Cells were grown in six-well cell culture plates and were treated with varying concentrations of LY5 for 6 hours. Viable cells were determined by trypan blue staining and counted. A low cell density of viable cells (10^4^/plate) were then seeded on 10 cm plates and continued to grow for two to three weeks. Cells were washed with PBS twice and fixed with cold methanol for 15 min. After fixation, cells were stained with 0.5% crystal violet (25% methanol) at room temperature for 10 min. After the staining, the plates were rinsed with distilled water and dried.

### Western blot analysis

Cancer cells were treated with LY5 (1.0, 2.5 μM, 5 μM) or DMSO for 24 hours. After the treatments, cells were collected. For IL-6 and IFN-γ stimulation experiments, DLD1 and Hep3B cells were serum-starved for 12 hours and pretreated with LY5 (1.0, 2.5 μM, and/or 5 μM), or DMSO for 2 hours. Then, 50 ng/ml IL-6 or IFN-γ was added, and the cells were harvested 30 minutes later. IL-6 and IFN-γ were purchased from Cell Signaling Technology. The collected cells were washed with cold PBS and lysed on ice in a modified RIPA buffer (1% Triton X-100, 1% deoxycholate, 0.1% SDS) containing protease inhibitors (1 mM PMSF), subjected to SDS-PAGE. Proteins were transferred onto PVDF membrane and probed with antibodies (Cell Signaling Tech.). Membranes were probed with a 1:1000 dilution of primary antibodies (Cell Signaling Tech.) against phospho-specific STAT3 (Tyrosine 705, #9131), phospho-independent STAT3 (#4904), phospho-specific ERK1/2 (Threonine 202/Tyrosine 204, #4370), phospho-specific Akt (Threonine 308, #9275), cleaved caspase-3 (Asp175, #9661), Bcl-2 (#2876), Suvivin, Bcl-xl (#2762), cyclinD1 (#2922) and GAPDH (#2118). HRP-conjugated secondary antibodies were from Santa Cruz Biotechnology. The specific proteins were detected using an enhanced chemiluminescence (ECL) Western Blotting kit according to the manufacturer's instructions.

### Immunofluorescence staining

Cells were seeded on sterile glass slides and grown for 24 hours. The next day, cells were treated with LY5 for 24 hours. For nuclear translocation experiment, after serum-free overnight Hep3B and DLD1 cells were pre-treated with LY5 for 2 hours, then IL-6 was added for another 30 minutes. After the treatments, the cells were washed with ice-cold phosphate-buffered saline (PBS) buffer, and were fixed with ice-cold methanol at room temperature for 15 minutes. After two washings with ice-cold PBS buffer, the cells were permeabilized and blocked with PBS buffer containing 0.3% Triton X-100 and 5% normal goat serum at room temperature for at least 1 hour. Then the cells were probed with the polyclonal rabbit antibody to phosphorylated STAT3 or total STAT3 (1:50 dilution, 1:100 dilution respectively) at 4°C overnight. After the over night incubation, the cells were washed with PBS buffer containing 0.1% Tween-20. The cells were incubated with FITC-conjugated anti-rabbit secondary antibody (Jackson ImmunoResearch Laboratories, West Grove, PA) or Alexa Fluor Dye (1:100, Alexa Fluor 594 goat anti-rabbit IgG) anti-rabbit secondary antibody (Molecule Probe, Invitrogen) at room temperature for 1 hour. The cells were mounted with VectashieldHardSet mounting medium with DAPI Vector Laboratories, Burlingame, CA. Pictures were captured by fluorescent microscope.

### Wound healing assay

Cells were seeded in 6-well cell culture plates with Dulbecco's modified Eagle's medium/high glucose containing 10% FBS. After the cells reached 100% confluence, the monolayer was scratched using a 10-ul pipette tip and washed once to remove non-adherent cells. Cells were treated with DMSO, 2.5 μmol/L, and 5 μmol/L of LY5 in the presence of 10% FBS for 2 hours. After that, the medium was replaced and fresh medium supplemented with 10% FBS was added. After additional 24–48 hours without LY5 treatment, the cells were observed under the microscope. When the wound in the DMSO control was closed, the inhibition of cell migration was assessed by using the ImageJ software, available from the NIH website (http://rsb.info.nih.gov/ij/). The % of wound healed was calculated using the formula: 100 - (final area/initial area × 100%).

### Mouse xenograft tumor model

Human colon cancer cell line, HCT116 (10^7^ cells in 100 μL of sterile PBS and matrigel), were injected subcutaneously into the right flank region of female athymic nude mice (4 to 6 weeks of age, 18–22 grams). After tumor development, mice were divided into two treatment groups (*n* = 6 each): (I) dimethyl sulfoxide (DMSO) as vehicle control and (II) 5 mg/kg of LY5 (dissolved in 10% DMSO, 18% Cremophor EL and 72% sterile 5% dextrose). Vehicle and LY5 were administered via intraperitoneal injection once daily for 11 days. Tumor growth was determined by measuring the length (L) and width (W) of the tumor every other day with a caliper. And tumor volume was calculated on the basis of the following formula: volume = (π/6)*L*W^2^. The tumors were harvested after mice was killed, snap-frozen in liquid nitrogen, and stored at −80°C. Tumors tissue homogenates were lysed and separated by SDS-PAGE. A portion of tumor tissues were fixed by using formalin and embedded in paraffin. The expression of STAT3, its downstream target genes Bcl-2 and cleaved caspase 3 in xenograft tumors was examined by Western Blot assay. The expression of P-STAT3 (Y705) and Bcl-2 was also examined by immunohistochemistry (IHC) staining. TUNEL (Terminal deoxynucleotidyl transferase dUTP nick end labeling) assay was used to detect the percentage of apoptosis in xenograft tumors.

### Statistical analysis

All of the data were presented as the mean ± SD for at least three independent experiments. Statistical analysis was performed with SPSS software (version 13.0). The significant differences between any of two groups were evaluated by One-way ANOVA. Statistical significance was defined as *P* <0.05.

## SUPPLEMENTARY MATERIALS FIGURES AND TABLES


