# Supplementary material for: A novel small molecule STAT3 inhibitor, LY5, inhibits cell viability, colony formation, and migration of colon and liver cancer cells
Source: Oncotarget. 2016 Feb 11;7(11):12917–26. doi: 10.18632/oncotarget.7338 (PMC4914331; doi:10.18632/oncotarget.7338)
Supplement: Supplementary file 1 [file oncotarget-07-12917-s001.pdf]

A novel small molecule STAT3 inhibitor, LY5, inhibits cell viability, colony formation, and migration of colon and liver cancer cells

Supplementary Materials

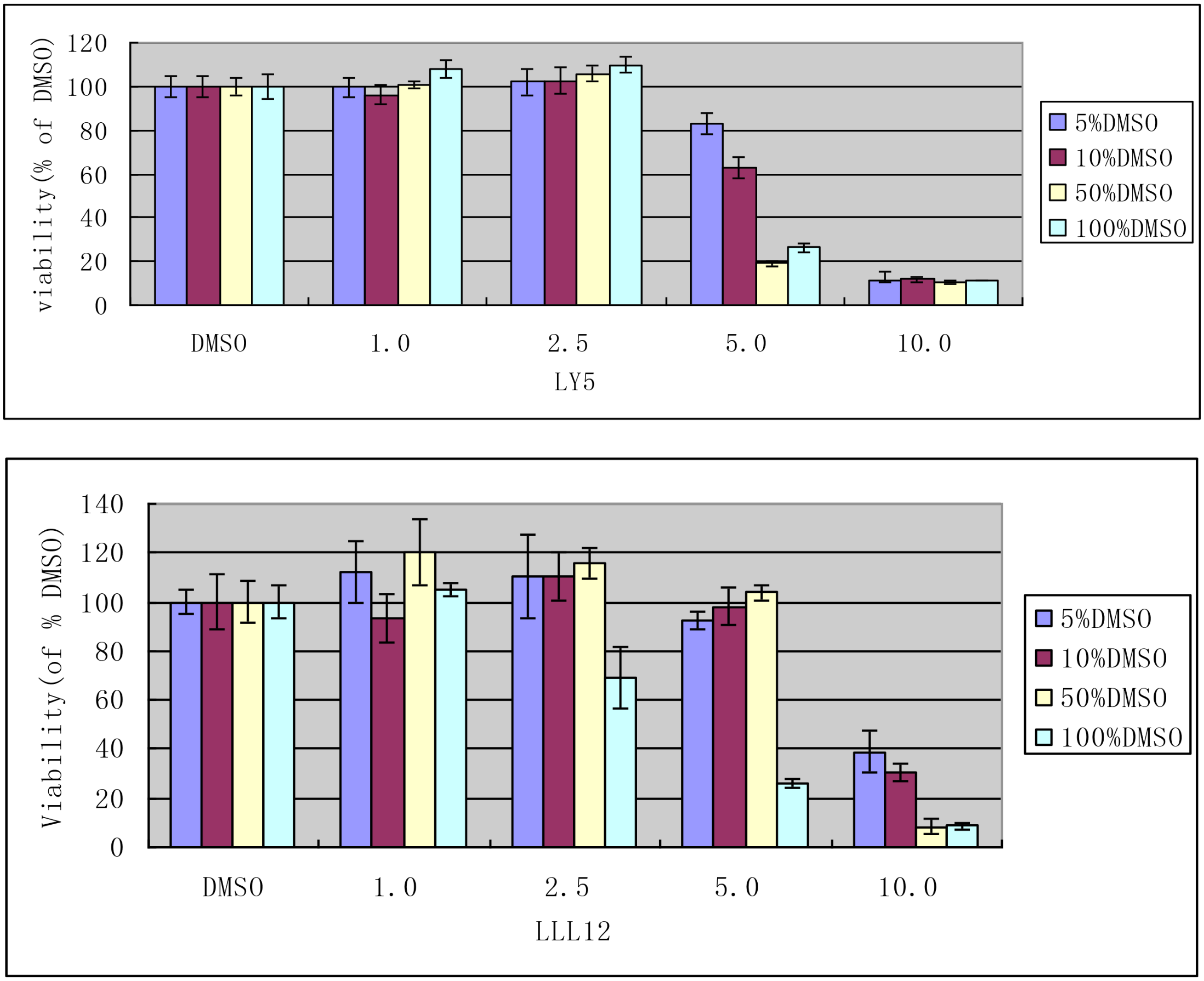

Supplementary Figure S1: Comparison of LY5 and LLL12 in inhibiting cell viability in Hep3B liver cancer cells.

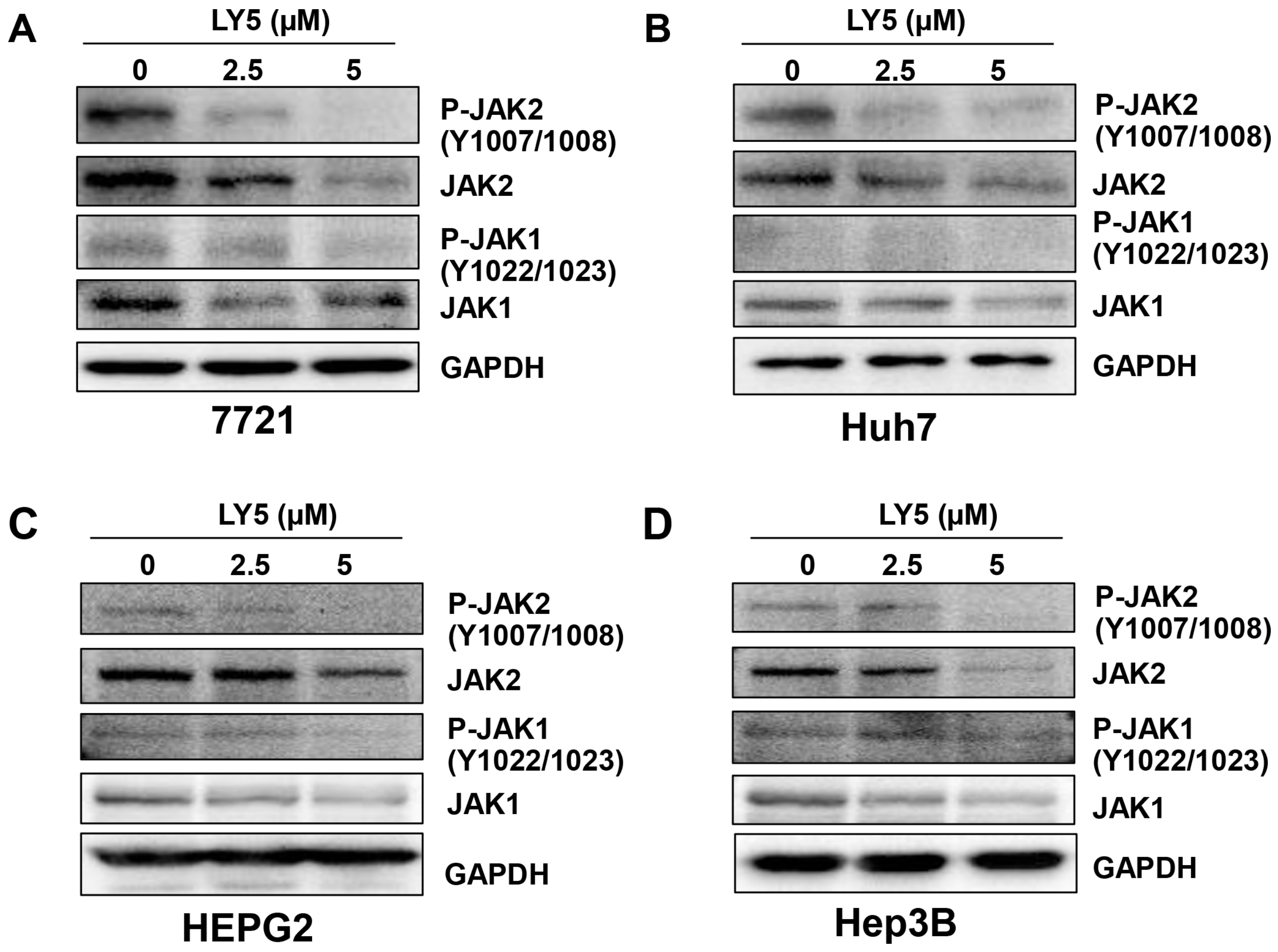

Supplementary Figure S2: The effects of LY5 on JAK1/P-JAK1 and JAK2/P-JAK2 in liver cancer cell lines.

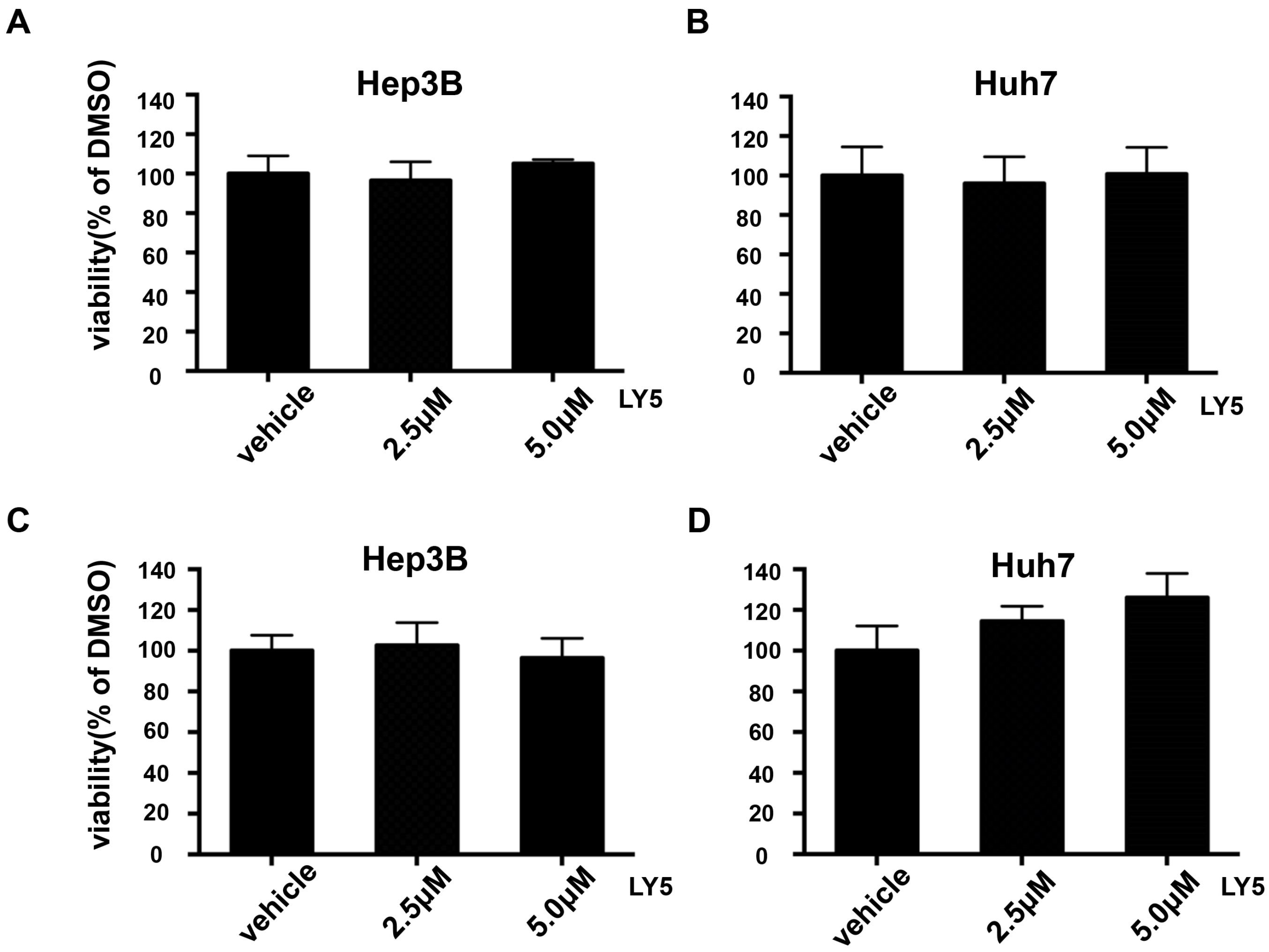

Supplementary Figure S3: The ability of LY5 to inhibit cell migration and colony formation is not due to induction of cell death. The time points of treatment with LY5 and incubation without LY5 used in wound healing assay and colony formation was applied in viability assay.

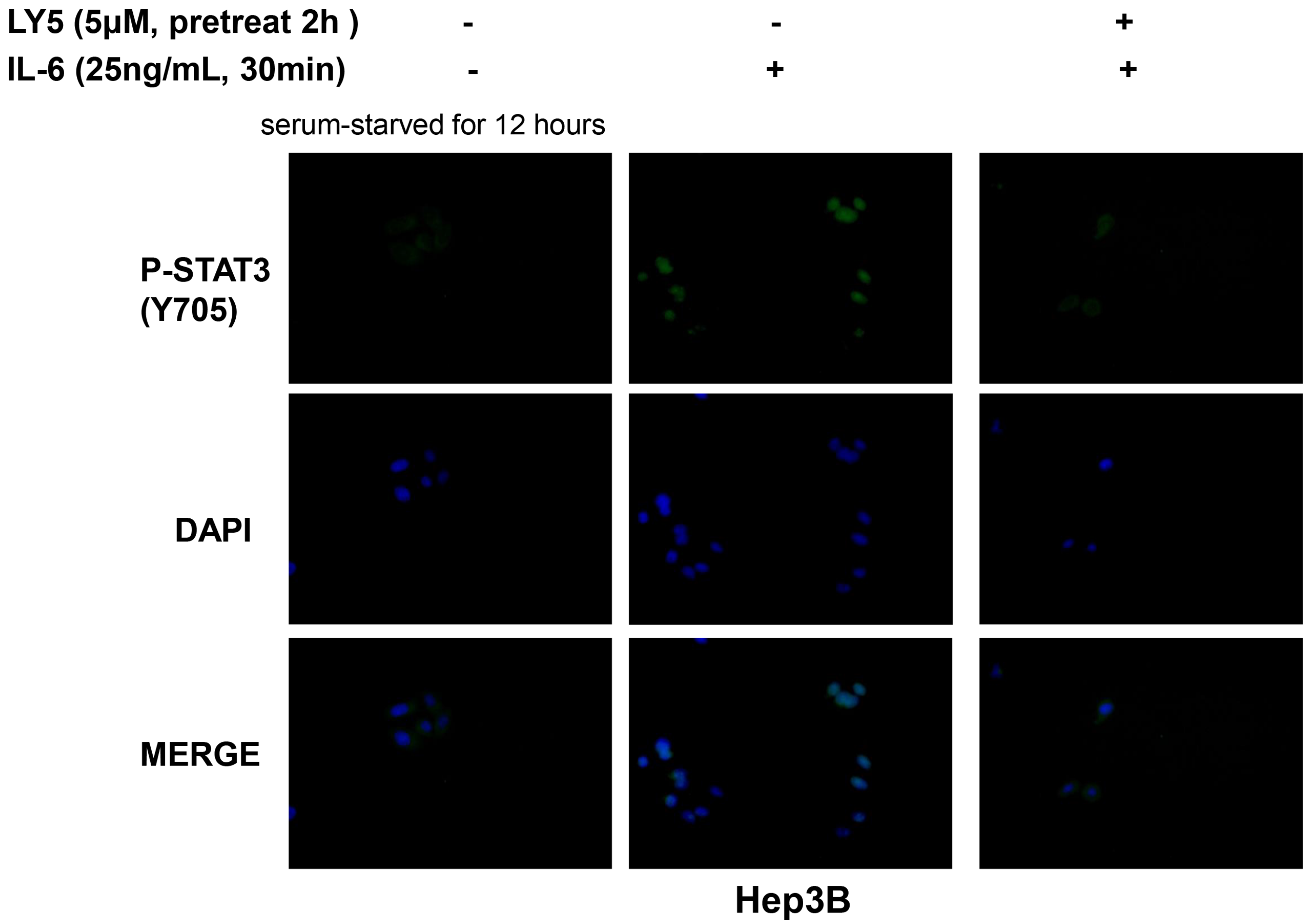

Supplementary Figure S4: LY5 inhibited STAT3 phosphorylation induced by cytokine IL-6 in Hep3B liver cancer cells. Hep3B liver cancer cells were plated on cover slides overnight and cultured in serum-free medium for 12 hours, then treated or untreated with the indicated concentrations of LY5. After 2 hours, the cells were stimulated by IL-6 (25 ng/mL) for 30 min, then analyzed for STAT3 phosphorylation by immunofluorescence staining as described in Materials and Methods.

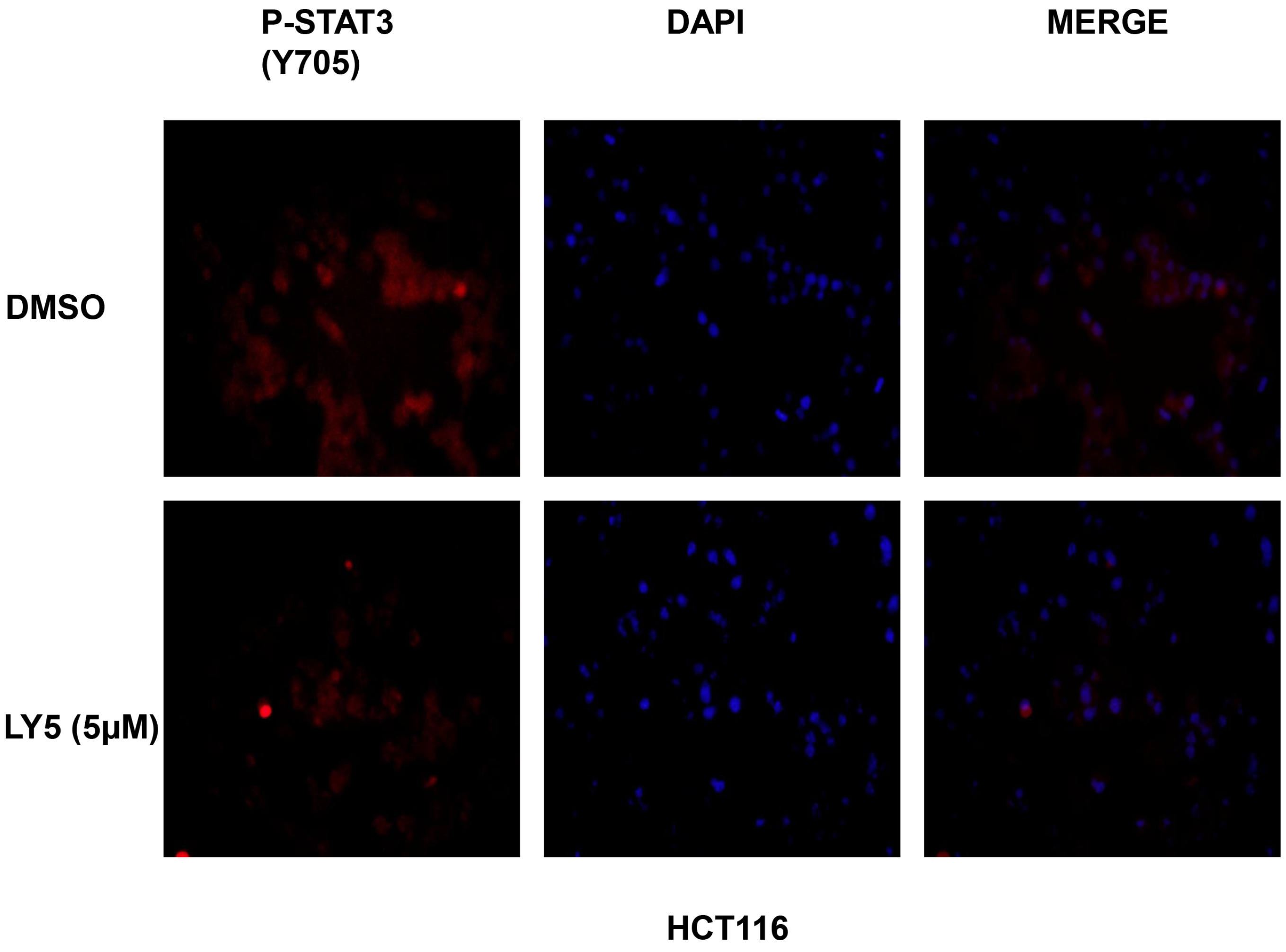

Supplementary Figure S5: LY5 inhibited STAT3 phosphorylation in the nuclei of HCT116 colon cancer cells.

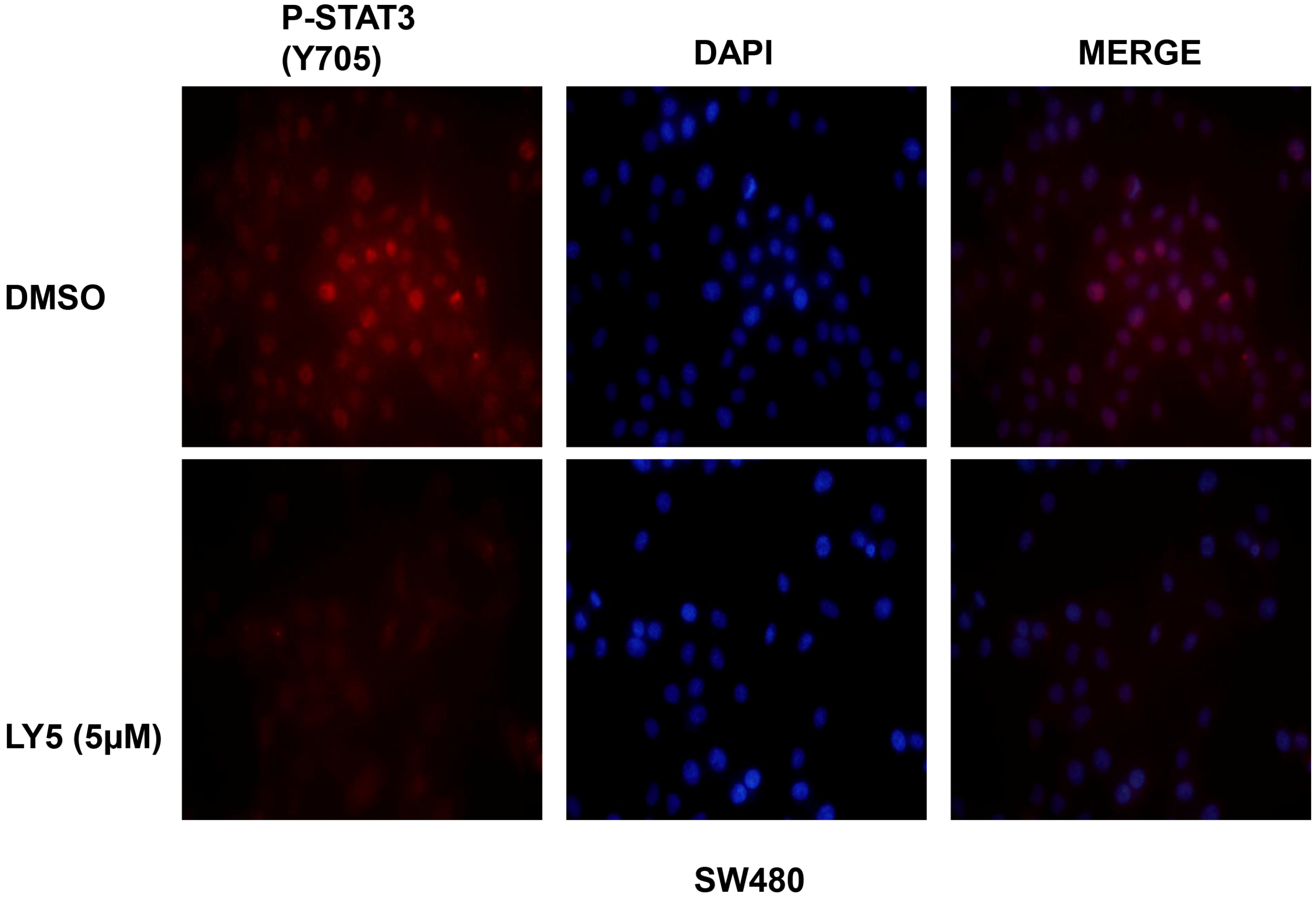

Supplementary Figure S6: LY5 inhibited STAT3 phosphorylation in the nuclei of SW480 colon cancer cells.

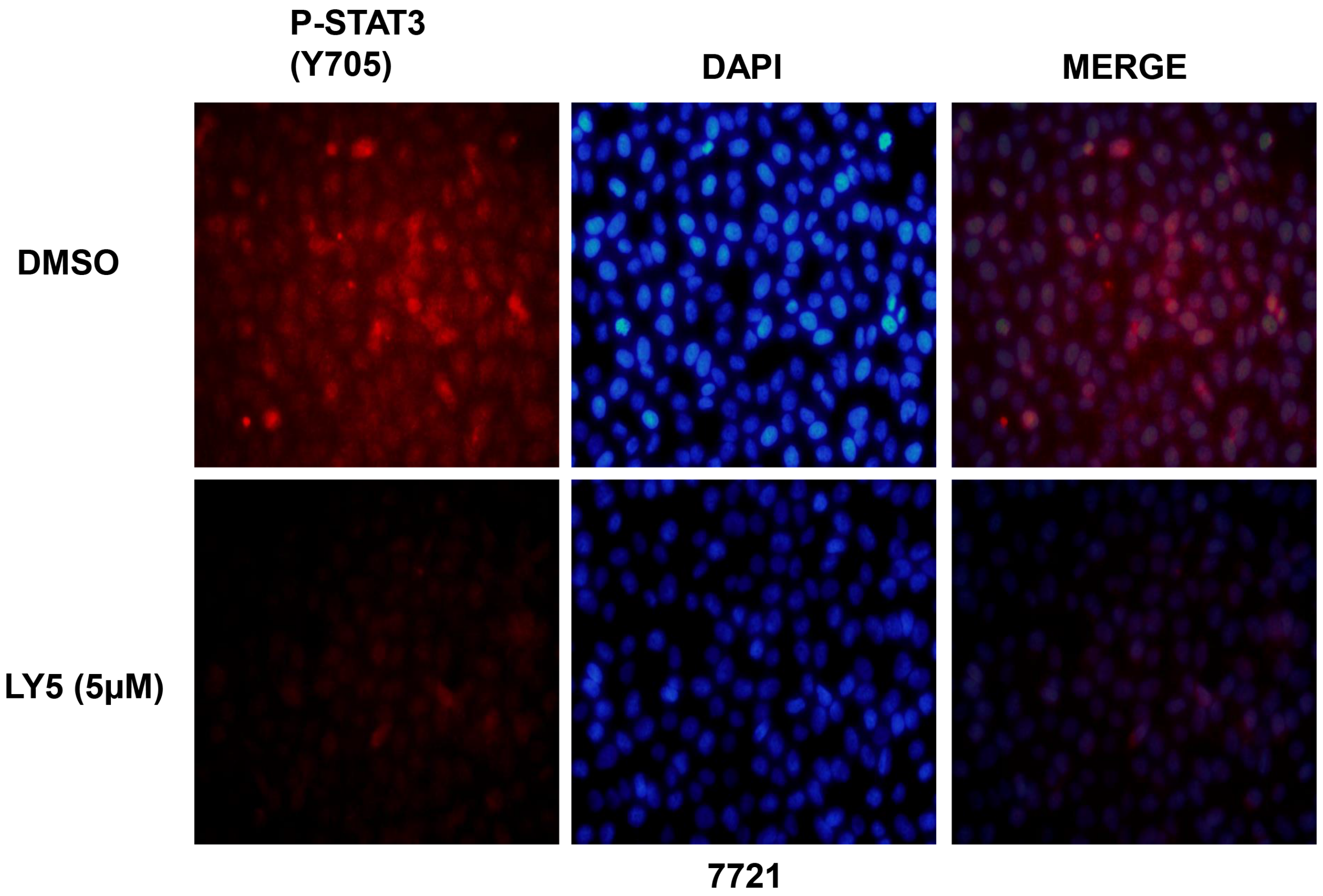

Supplementary Figure S7: LY5 inhibited STAT3 phosphorylation in the nuclei of 7721 liver cancer cells.

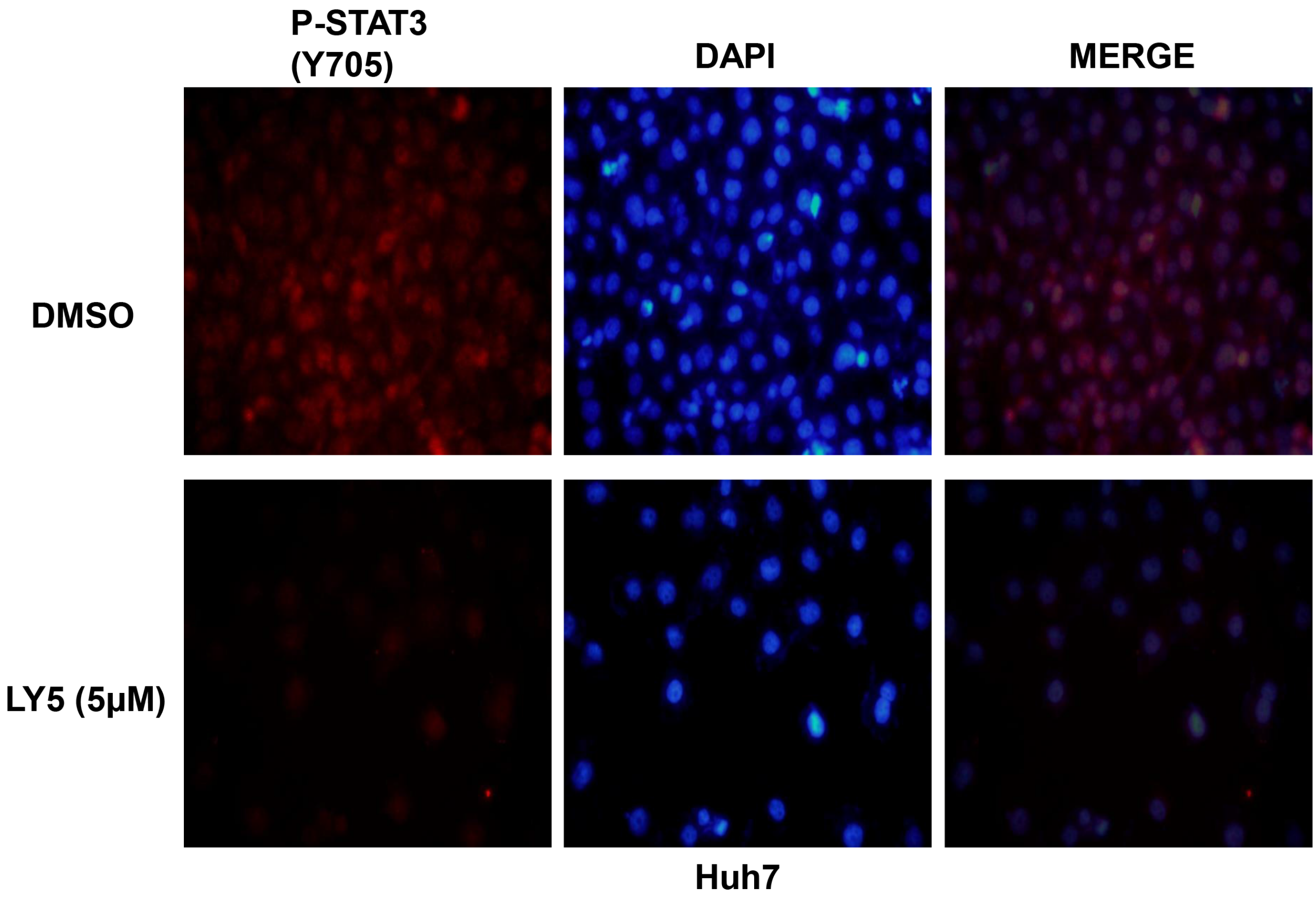

Supplementary Figure S8: LY5 inhibited STAT3 phosphorylation in the nuclei of Huh7 liver cancer cells.

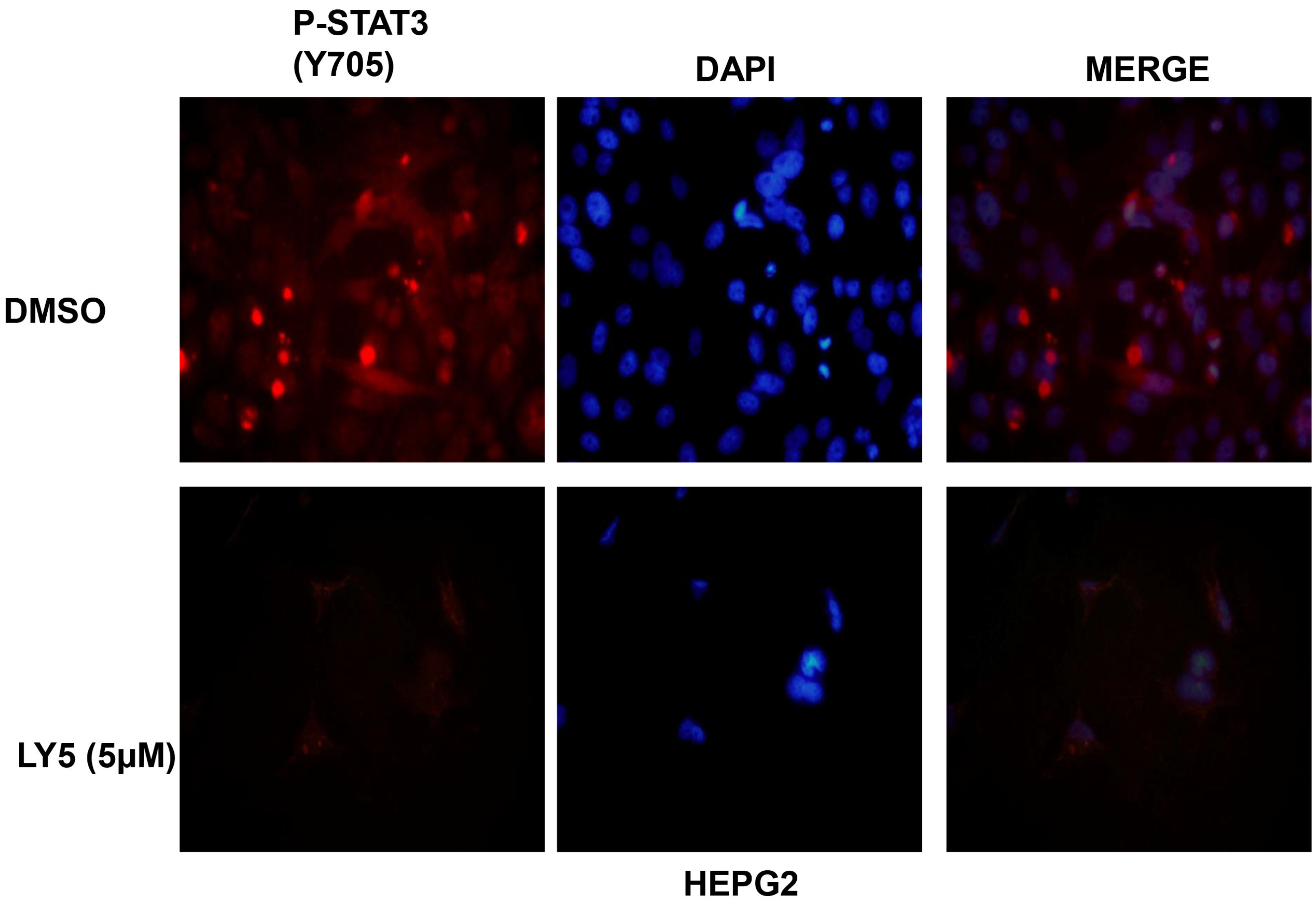

Supplementary Figure S9: LY5 inhibited STAT3 phosphorylation in the nuclei of HEPG2 liver cancer cells.

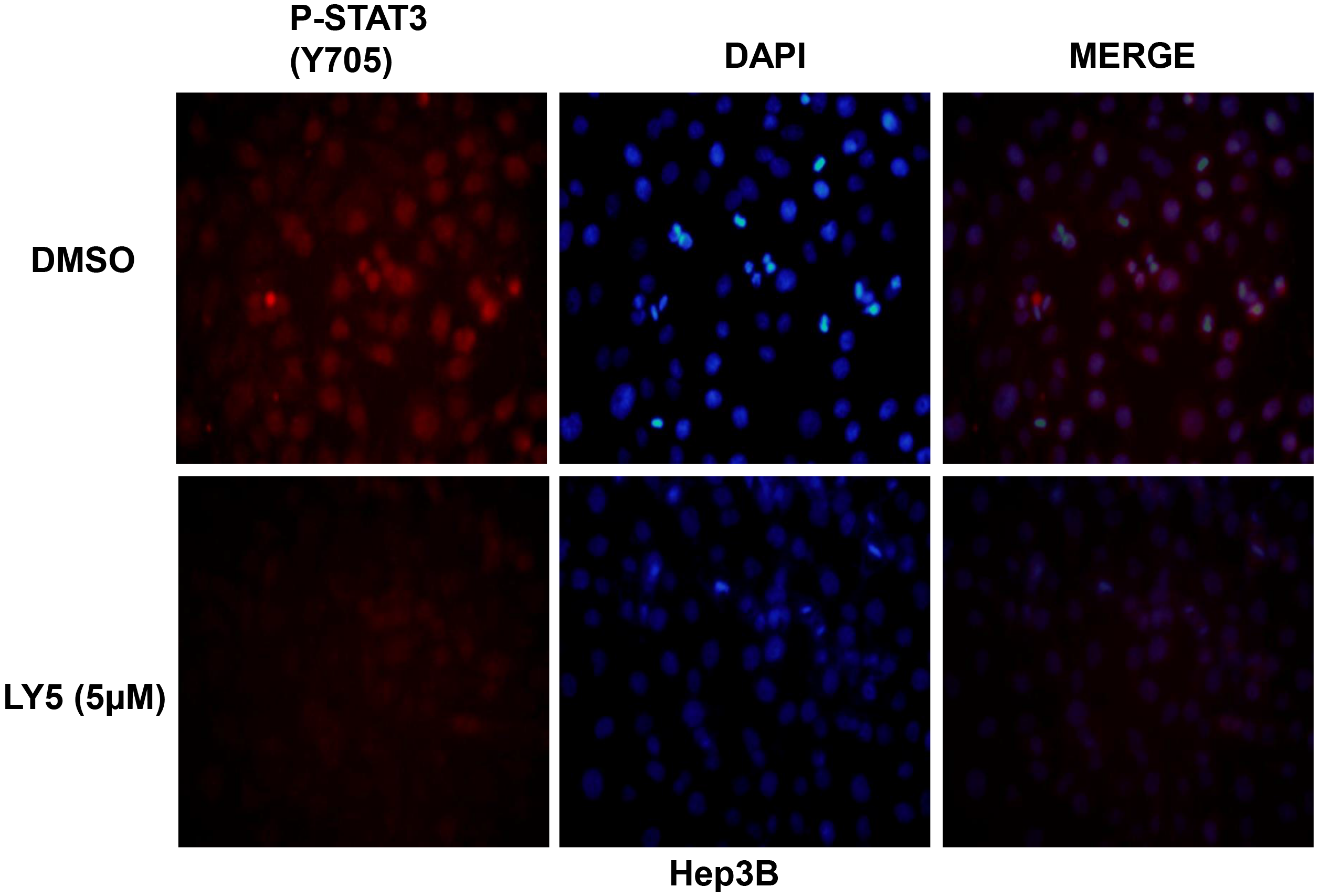

Supplementary Figure S10: LY5 inhibited STAT3 phosphorylation in the nuclei of Hep3B liver cancer cells.

Supplementary Table S1: IC50 (μM) of LLL12 and LY5 in Hep3B liver caner cells

DMSO	LLL12	LY5
5%	9.054	6.345
10%	9.11	5.02
50%	7.146	1.242
100%	3.8	2.539

LLL12 and LY5 were dissolved in 5, 10, 50, and 100% DMSO respectively.

Supplementary Table S2: IC50 (μM) of LY5 in human liver and colon caner cells

	Human liver caner cells			Human colon caner cells		
	Hep3B	Huh7	7721	HCT116	DLD1	SW480
LY5	3.263	1.637	3.347	1.690	1.370	1.235
